# Predicting motor, cognitive & functional impairment in Parkinson's

**DOI:** 10.1002/acn3.50853

**Published:** 2019-07-26

**Authors:** Christine Lo, Siddharth Arora, Fahd Baig, Michael A. Lawton, Claire El Mouden, Thomas R. Barber, Claudio Ruffmann, Johannes C. Klein, Peter Brown, Yoav Ben‐Shlomo, Maarten de Vos, Michele T. Hu

**Affiliations:** ^1^ Oxford Parkinson's Disease Centre (OPDC) University of Oxford Oxford UK; ^2^ Nuffield Department of Clinical Neurosciences University of Oxford Oxford UK; ^3^ Somerville College, University of Oxford Oxford UK; ^4^ Population Health Sciences University of Bristol Bristol UK; ^5^ Medical Research Council Brain Network Dynamics Unit University of Oxford Oxford UK; ^6^ Institute of Biomedical Engineering University of Oxford Oxford UK

## Abstract

**Objective:**

We recently demonstrated that 998 features derived from a simple 7‐minute smartphone test could distinguish between controls, people with Parkinson's and people with idiopathic Rapid Eye Movement sleep behavior disorder, with mean sensitivity/specificity values of 84.6‐91.9%. Here, we investigate whether the same smartphone features can be used to predict future clinically relevant outcomes in early Parkinson's.

**Methods:**

A total of 237 participants with Parkinson's (mean (SD) disease duration 3.5 (2.2) years) in the Oxford Discovery cohort performed smartphone tests in clinic and at home. Each test assessed voice, balance, gait, reaction time, dexterity, rest, and postural tremor. In addition, standard motor, cognitive and functional assessments and questionnaires were administered in clinic. Machine learning algorithms were trained to predict the onset of clinical outcomes provided at the next 18‐month follow‐up visit using baseline smartphone recordings alone. The accuracy of model predictions was assessed using 10‐fold and subject‐wise cross validation schemes.

**Results:**

Baseline smartphone tests predicted the new onset of falls, freezing, postural instability, cognitive impairment, and functional impairment at 18 months**.** For all outcome predictions AUC values were greater than 0.90 for 10‐fold cross validation using all smartphone features. Using only the 30 most salient features, AUC values greater than 0.75 were obtained.

**Interpretation:**

We demonstrate the ability to predict key future clinical outcomes using a simple smartphone test. This work has the potential to introduce individualized predictions to routine care, helping to target interventions to those most likely to benefit, with the aim of improving their outcome.

## Introduction

Significant heterogeneity in Parkinson's influences clinical presentation, progression, medication response, and disease complication risk. The Oxford Discovery and Tracking Parkinson's cohorts provide around 2500 community‐ascertained patients, prospectively followed from early diagnosis, in whom these phenotypic variations can be studied.^1^ We used data‐driven approaches to identify fast and slow motor progressor subtypes, with differences akin to the minimally clinically important 3‐point difference on the Movement Disorders Society Unified Parkinson's Disease Rating Scale (MDS‐UPDRS) that neuroprotective treatment trials are often powered to detect.^1^, ^2^, ^3^ While such differences are observed in cohort studies, individualized predictions remain challenging.

Disability in Parkinson's is mainly determined by the onset of postural instability, falls, freezing of gait, and dementia.^4^, ^5^ The time to reach these disease milestones varies considerably, leading to increased outcome variation and requiring larger sample sizes to demonstrate potential treatment effects.^6^ A number of models have aimed to predict these clinically relevant outcomes. A 3‐step falls prediction tool by Paul et al. attached the greatest weighting to whether individuals reported falling at baseline, yet limited numbers prevented the prediction of the onset of falls in those without falls at baseline, an outcome of greater interest to the treating clinician.^7^, ^8^

Ehgoetz et al. recently reported a logistic regression model utilizing the Hospital Anxiety and Depression Scale (HADS) and the Freezing of Gait (FOG) questionnaire total score to predict the onset of future freezing, which requires external validation.^5^ Velseboer et al. described a similar model utilizing age, the numbers of animals named in a verbal fluency task and the UPDRS axial score to predict a composite adverse outcome but it was not possible to distinguish between death, dementia or postural instability.^9^ Models to date have relied on combinations of different clinical questionnaires and assessments, requiring time and skill to administer, in order to make specific predictions; to the best of our knowledge no single test has been able to predict multiple future clinical outcomes.

A multi‐device study is being planned, that uses smartphones to capture questionnaire data and to store tremor data recorded by smartwatches, alongside tablet‐based assessments, with the aim of differentiating Parkinson's from Essential Tremor.^10^ However so far, studies using smartphones alone have focused on equipping the clinician; in distinguishing individuals with and without Parkinson's^11^, ^12^ and working to derive smartphone scales with which to measure disease severity.^13^, ^14^ Our aim was to use smartphone data to predict outcomes of direct clinical relevance to people with Parkinson's and clinicians, alike.

## Methods

The Oxford Parkinson's Disease (PD) Centre Discovery study^15^ is a longitudinal cohort study that recruits participants with early Parkinson's who fulfil the United Kingdom PD Brain Bank criteria for probable PD.^16^ Continued participation depends upon participants being ascribed a probability of Parkinson's of at least 90% by trained researchers at their latest clinic assessment.^17^ Antecedent approval was granted by the local research ethics committee, adhering to national legislation and the Declaration of Helsinki. Participants provide written informed consent to participate.

**Figure 1:**
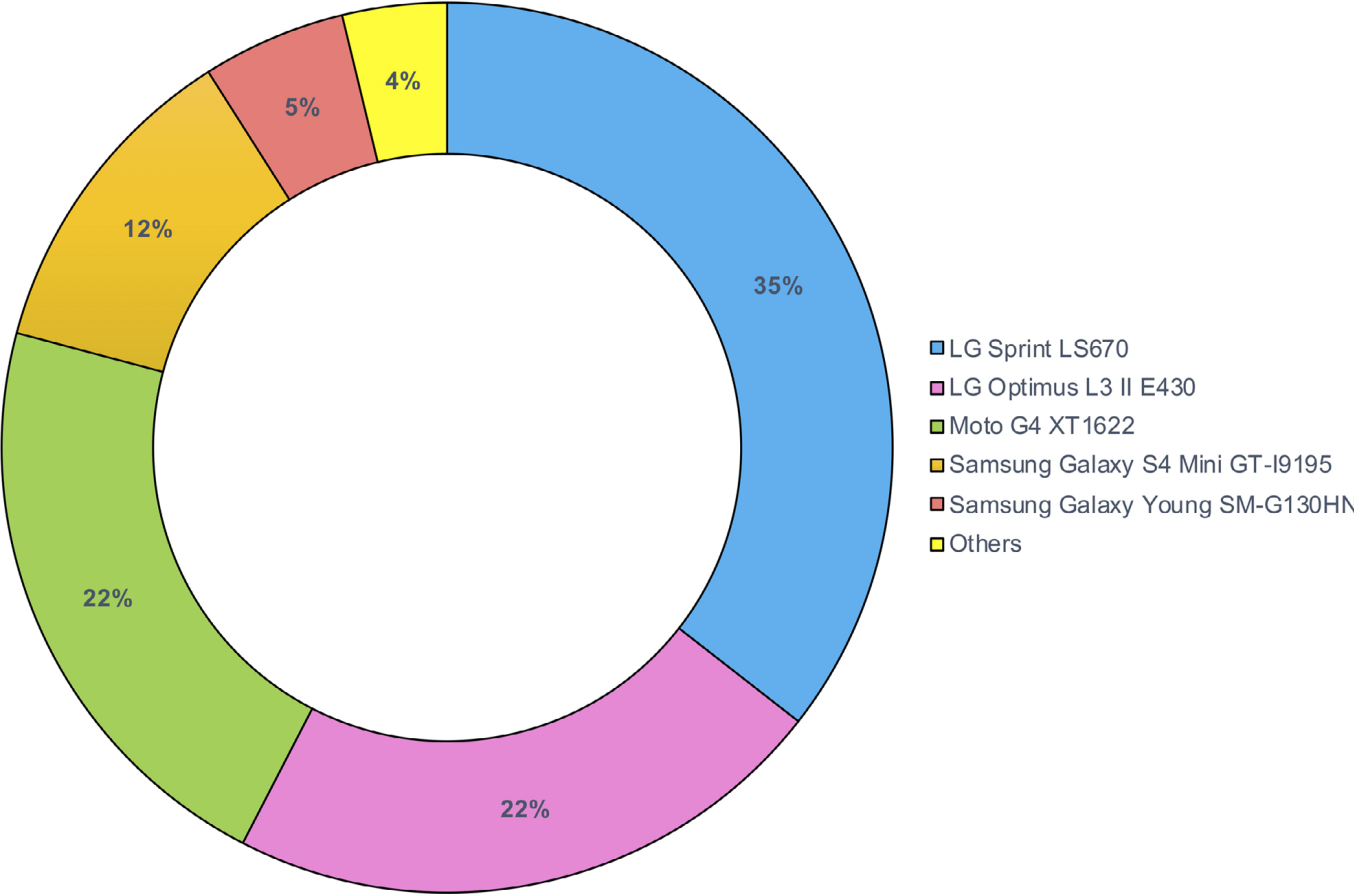
Smartphone models. In the search for a scalable solution to the quantification of motor symptoms in Parkinson's, an Android based smartphone app was installed on a range of consumer grade smartphones that were used in clinic and provided to participants to take home. Participants also had the option of being sent a link to download the app onto their own Android smartphone. A specialized smartphone app was used to collect the raw accelerometer, microphone and screen data and was run alongside KitKat, Lollipop, Marshmallow, Nougat, and Oreo Android operating systems. The raw data from the app was encrypted, time‐stamped, and uploaded to a secure online server. The processing and analysis of the data was performed separately using computer‐based Matlab® software (R2018a; Mathworks®, USA). “Others” include: Samsung Galaxy Ace 4 SM‐G357FZ, Samsung Galaxy Ace 2 GT‐I8160, Samsung Galaxy S3 Mini GT‐I8200N, LG Optimus 3G CX670, Samsung Galaxy S III mini I8190, Samsung Galaxy J5 J500FN, Sony Xperia L C2105, Moto G LTE XT1039, Huawei Ascend G510, Samsung Galaxy S4 I9505.

Since 2014, participants have been invited to perform smartphone assessments. We analysed smartphone tests performed at 18‐monthly clinic visits and up to four times a day for a maximum of seven days at home, within a 3‐month period of their clinic visit (see Fig. 1 for device details).^18^ Smartphone tests assess: (1) Voice (participants hold the phone to their ear, take a deep breath, and say “aaah” at a comfortable and steady tone and level, for as long as possible); (2) Balance and (3) Gait (with the phone in a trouser pocket or arm band, participants stand still and then walk a distance of 20 yards before turning and walking back); (4) Dexterity (participants tap alternately between two buttons on the screen at a comfortable rate); (5) Non‐cued reaction time (participants press on a button as it appears on the screen, keeping their finger down whilst it is there and lifting their finger off as it disappears); (6) Rest and (7) Postural tremor (participants hold the smartphone in the hand most affected by tremor if they have tremor, or their dominant hand if they do not, while their hand is at rest or held outstretched in front of them).

The seven smartphone tasks take 6–7 min in total to perform. All seven tasks constitute one smartphone recording. Incomplete recordings, where all seven tasks were not performed within a 15‐min time period, were excluded from analysis.

Clinical data collected at in‐person 18‐monthly longitudinal clinic visits were matched to smartphone recordings performed in clinic and at home within 3 months of the clinic visit; henceforth referred to as a time window. Smartphone recordings contributed at different time windows, related to different longitudinal clinic visits, were treated independently for the purpose of analysis. Smartphone recordings analyzed were collected between 8 August 2014 and 7 November 2017.

Future outcomes were defined according to the results of clinical assessments and questionnaires performed at the next 18‐month clinic visit as detailed in (Table 1) and included the new onset of (1) falls (>1 self‐reported fall in the preceding 6 months), (2) freezing (a freezing frequency of at least “about once a month” on the FOG questionnaire^19^), (3) Postural instability (Hoehn and Yahr^20^ stage ≥ 3), (4) Cognitive impairment^21^ (a Montreal Cognitive Assessment (MoCA)^22^ score < 26), (5) Difficulty doing hobbies (MDS‐UPDRS^23^ part II item 2.8 score ≥ 3 indicating major difficulty or an inability to do activities of enjoyment) and (6) the self‐reported need for future help at home. Individuals who had already reached a given milestone at the time of their smartphone recording were excluded from the respective analysis. Similarly, smartphone recordings missing the clinical data necessary for the assessment of the outcome definition or meeting exclusion criteria were excluded. All assessments were done on existing medication.

**Table 1:** Smartphone predictions: outcome definitions and exclusion criteria.

Future outcome in 18 months' time	Clinical tool	Baseline exclusion	Outcome definition
Falls	Falls questionnaire Frequency of self‐reported falling	*Exclusion of those already falling* [at least 1 fall in the preceding 6 months]	*The onset of falls* [at least one fall in the 6 months preceding their next 18‐month visit]
Freezing	Freezing of gait questionnaire^18^: Question 3: “Do you feel that your feet get glued to the floor while walking, making a turn or when trying to initiate walking (freezing)?”	*Exclusion of those already freezing *[a score ≥ 1 i.e. any answer other than “Never”]	*The onset of freezing *[a score ≥ 1 in 18 months' time]
Postural instability	Hoehn and Yahr^19^	*Exclusion of those with existing postural instability* [a stage ≥ 3]	*The onset of postural instability* [a stage ≥ 3 in 18 months' time]
Cognitive impairment	Montreal Cognitive Assessment^21^	*Exclusion of those with existing evidence of cognitive impairment* [a score < 26]	*The onset of cognitive impairment* [A MoCA < 26 in 18 months' time]^a^
Difficulty doing hobbies	Movement Disorders Society‐Unified Parkinson's Disease Rating Scale part II^22^: Question 2.8: “Over the past week, have you usually had trouble doing your hobbies or other things that you like to do?”	*Exclusion of those with existing moderate to severe difficulty doing hobbies or other activities of enjoyment* [a score ≥ 3]	*The onset of major problems or an inability to do hobbies or other activities of enjoyment* [a score ≥ 3 in 18 months' time]
Need for help at home	Social background questionnaire: Self‐reported need for help at home	*Exclusion of those who are already needing help at home*	*The onset of a need for help at home*

Abbreviation: MoCA, Montreal Cognitive Assessment.

a A MoCA score < 26 has previously been found to be associated with a sensitivity of 86% and specificity of 72% in screening for individuals with deficits on neuropsychological testing in at least two domains.^33^

Altogether 998 statistical features were extracted from each smartphone recording as previously described.^24^ These features help characterize different motor symptoms associated with Parkinson's. Typical examples of such features include variation in speech frequency and amplitude, degree of turbulent noise in speech due to incomplete vocal fold closure, tapping speed and rhythm, mean reaction time, changes in body motion (such as detrended fluctuation analysis) and association between the tri‐axial accelerometer sensor data (such as mutual information). Statistical analyses were performed using Matlab® software (R2018a; Mathworks®, USA).

Smartphone features were used to train machine learning algorithms (random forests) to predict each binary clinical outcome of interest. Random forests is an ensemble learning technique that has commonly been used for a range of classification and regression applications.^25^ We chose random forests as they are relatively robust to outliers and noisy features. Moreover, in previous smartphone‐based studies, random forests have been shown to be useful in distinguishing participants with RBD and Parkinson's from controls,^18^ and in predicting MDS‐UPDRS part III motor examination scores.^12^ To try and avoid model overfitting, we used cross validation (CV) which involves splitting the dataset into non‐overlapping training and test sets. The training data was used for learning the underlying patterns of interest; the test dataset for validating the model prediction accuracy. CV helps assess the generalizability of the model to previously unseen datasets. To test the accuracy of predictions, we used two common methods of CV: (1) 10‐fold CV, and (2) leave one subject out (LOSO) CV.

Machine learning is also sensitive to the proportion of data with and without the clinical outcome of interest that is used to train the model. If there is an imbalance in outcome between groups, for example, where significantly more recordings from people without falls are used to train a falls prediction model, high prediction accuracies may simply reflect the prevalence of the outcome of interest in the dataset. Data were therefore balanced in a 1:1 ratio prior to the training of algorithms for both CV schemes by randomly under‐sampling the majority class.

For 10‐fold CV, to balance the dataset, an equal randomly selected sample of recordings from participants without the outcome of interest was merged with recordings with the outcome of interest, assuming independence between recordings and ignoring within person correlations. The recordings were randomized and partitioned into 10 separate folds. In turn, each fold was set aside to form the test set, on which the accuracy of the model, trained using the remaining mutually exclusive 9 folds, was tested. Ten repetitions of 10‐fold CV, each with randomly selected balanced recordings, were performed. Similarly, datasets were randomly balanced by outcome for LOSO CV, where only the single clinic recording performed by participants at their clinic visit was analysed; all home recordings were excluded. Home recordings were excluded from LOSO CV in order to allow for a direct comparison to be made with existing logistic regression models (detailed below); a single set of clinical data was matched to each smartphone recording performed in clinic for LOSO CV, with the clinical data also being fed into existing logistic regression models.^5^, ^9^ LOSO CV involves each recording in turn forming the test set, with the rest being used to train the model. Ten repetitions of LOSO CV, each with randomly selected balanced datasets, were performed.

Following both CV schemes (see Table S1 for description of modelling algorithm), the predicted probability of each recording belonging to either group was compared to the known future outcome. To summarize the accuracy of predictions we calculated area under the curve (AUC) values.

A level of redundancy is likely among the 998 smartphone features, with some being more important for the prediction of certain outcomes than others. Feature ranking was obtained using random forests (employing only the training data that was balanced at each LOSO CV iteration). The assigned predictor importance was averaged across all trained models for a given outcome prediction, to allow the ranking of features in order of importance. We then trained models using the top 30 smartphone features.

In order to gauge the validity of our machine learning approach and the strength of our smartphone features, we applied two existing logistic regression prediction models to our clinical data and assessed the accuracy of their predictions of (1) future freezing^5^ and (2) a composite future adverse outcome of dementia, postural instability, or death^9^ against those made using smartphone features, random forests and LOSO CV. Following personal communication with an author of the paper, direct MDS‐UPDRS values for items 3.9, 3.13, 3.10, and 3.12 were substituted for UPDRS items 27‐30 to form the axial score. Future freezing and postural instability were defined as before (Table 1). Dementia was defined according to the presence of both objective cognitive impairment (MoCA ≤ 20) as well as functional impairment (Informant Questionnaire on Cognitive Decline in the Elderly (IQCODE)>52).^21^, ^26^

## Results

**Figure 2:**
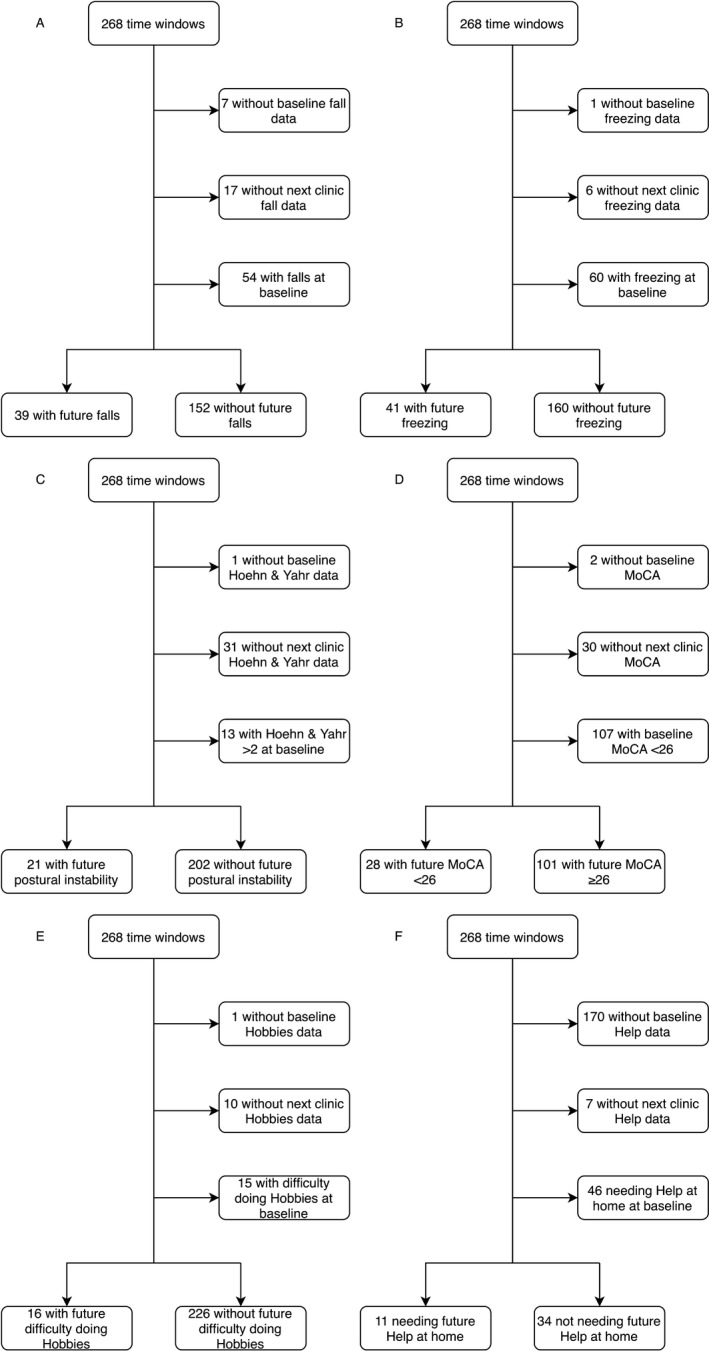
Flow charts demonstrating the time windows whose recordings were included in the analyses of the future onset of (A) falls, (B) freezing, (C) postural instability, (D) cognitive impairment, (E) difficulty doing hobbies, and (F) need for help at home.

A total of 1842 recordings performed by 237 participants at 268 time windows were included in the analysis of one or more outcomes (Fig. 2).

### Clinical characteristics

Baseline clinical characteristics of participants at the time of their smartphone recordings are presented in (Table 2). Participants who developed falls, postural instability, and cognitive impairment were significantly older than those who did not. Participants who developed falls and freezing had a longer disease duration from diagnosis and higher motor examination scores on the MDS‐UPDRS part III. Those who developed postural instability and difficulty doing hobbies had lower MoCA scores and higher MDS‐UPDRS part III scores compared to those who did not develop such future difficulty.

**Table 2:** Group characteristics at the time of the smartphone recordings.

Baseline data	Future prediction																	
	Falls			Freezing			Postural Instability			Cognitive impairment			Difficulty doing hobbies			Need for help		
Group	Falls	No falls	*P* 1	Freezing	No freezing	*P* 1	Postural instability	No postural instability	*P* 1	Cognitive impairment	No cognitive impairment	*P* 1	Difficulty doing hobbies	No difficulty doing hobbies	*P* 1	Need for help	No need for help	*P* 1
No. of participants	39	140	‐	41	145	‐	21	179	‐	28	82	‐	16	204	‐	11	28	‐
No. of time windows^2^	39	152	‐	41	160	‐	21	202	‐	28	98	‐	16	226	‐	11	33	‐
Mean (SD) Age at visit	71.9 (8.5)	66.6 (9.3)	0.00	69.9 (9.4)	67.6 (8.7)	0.14	74 (7.7)	67.6 (9)	0.00	70.0 (7.5)	64.8 (9.6)	0.00	70.2 (9.2)	68 (9.3)	0.35	69.0 (8.4)	64.2 (10.5)	0.18
No. (%) male	27 (69)	92 (61)	0.32	25 (61)	104 (65)	0.63	18 (86)	125 (62)	0.03	18 (64)	53 (54)	0.34	13 (81)	140 (62)	0.12	6 (55)	17 (52)	0.86
Mean (SD) duration (years) from diagnosis to visit	4.1 (1.9)	3.2 (2.2)	0.02	4 (2.2)	3.1 (2)	0.02	4.2 (1.8)	3.4 (2.2)	0.09	3.7 (2.4)	3.7 (2.4)	0.93	3.5 (2.1)	3.5 (2.2)	0.99	4.0 (1.6)	4.3 (2.4)	0.73
Mean (SD) levodopa dose equivalent	510.8 (243.1)	410.4 (288.4)	0.05	474.3 (278)	402.5 (257.8)	0.12	586.8 (359.6)	431.3 (286.6)	0.02	441.8 (272.6)	462.7 (293.8)	0.74	525.9 (293.7)	444 (288.2)	0.27	457.0 (250.1)	427.9 (264.3)	0.75
Mean (SD) MDS‐UPDRS III	31.2 (13.2)	26.2 (11.2)	0.02	31.2 (13.2)	25.6 (10.3)	0.00	33.7 (11.2)	26.5 (10.7)	0.00	27.6 (8.8)	24.3 (11.1)	0.16	35 (16.3)	27 (11.1)	0.01	24.1 (16.0)	23.5 (9.3)	0.88
Mean (SD) MoCA	24.9 (3.6)	25.4 (3.4)^3^	0.31	25.6 (3.8)	25.4 (3.5)^4^	0.79	23.2 (5.2)	25.4 (3.5)^5^	0.00	27.4 (1.3)	28.0 (1.4)	0.04	23.4 (2.7)	25.4 (3.3)^6^	0.01	25.4 (3.3)	26.9 (2.4)	0.10
Mean (SD) number of recordings per participant	6 (9.9)	7 (10.6)	0.59	9.5 (12)	6.9 (10.5)	0.18	2.5 (5.5)	7.6 (11)	0.04	8.7 (11.4)	6.8 (10.3)	0.42	6.5 (11.3)	7 (10.6)	0.84	10.1 (11.7)	7.5 (10.8)	0.50

Abbreviations: MoCA, Montreal Cognitive Assessment, MDS‐UPDRS, Movement Disorders Society‐Unified Parkinson's Disease Rating Scale.

1 *P*‐value determined using a two‐sample *t*‐test or chi squared test to compare those with and without the future outcome of interest.

2 Data were balanced as described in the methods section, prior to training of machine learning algorithms, treating time windows independently.

3 A MoCA score was not available for two participants within the group that did not develop falling,

4 Two participants who did not develop freezing,

5 Two participants who did not develop postural instability and

6 Two participants who did not develop difficulty with hobbies in the future. The means/SDs for the aforementioned subgroups were calculated across participants for whom data was available.

### Model validation

**Figure 3:**
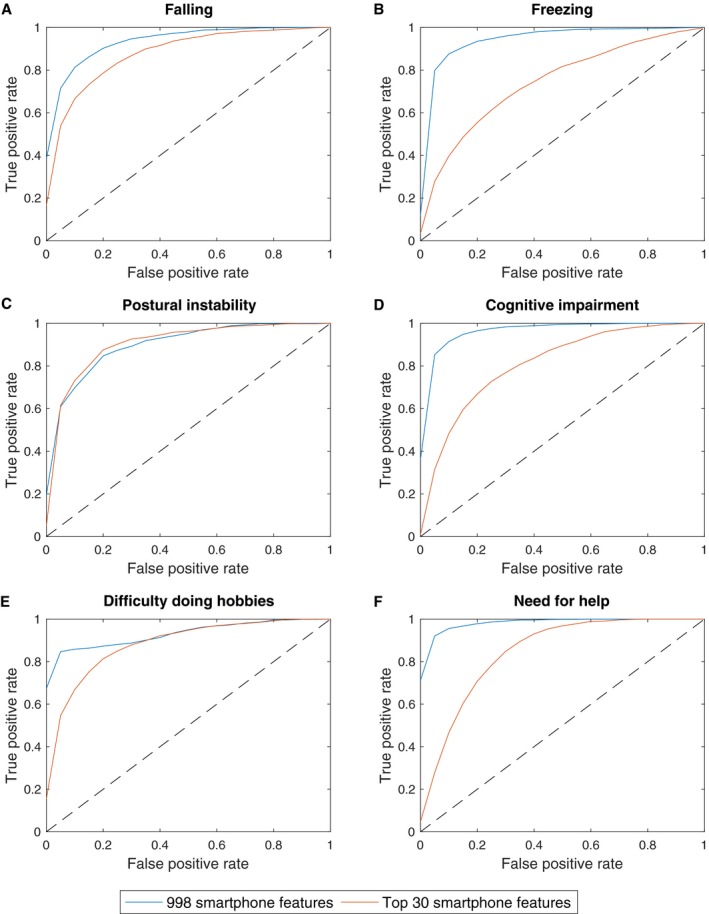
Receiver operating characteristic curves for classification by random forests in the prediction of the future onset of (A) falling, (B) freezing, (C) postural instability, (D) cognitive impairment, (E) difficulty doing hobbies and (F) the need for help. The diagonal dotted line corresponds to an AUC of 0.50 and indicates an uninformative model. The false positive rate (1‐specificity) is shown on the x axis and the true positive rate (sensitivity) is shown on the y axis.

It is likely that of the 998 smartphone derived features, some may be collinear (redundant) and associated with noise. Different sets of feature rankings were thus obtained separately for each of the outcomes of interest. Although multicollinearity can influence model inference, it does not reduce the overall predictive accuracy of the model. The results are displayed in (Fig. 3) and (Table 3). Using all 998 features, AUC values of greater than 0.90 were achieved for all six outcomes. Using only the top 30 smartphone features, with the highest predictive power, a reduction in the AUC values was seen though they remain greater than 0.75 for both 10‐fold CV and LOSO CV. The effect of increment in feature number on prediction accuracy (for 10‐fold CV and LOSO CV) is demonstrated in Figure S1.

**Table 3:** Results of random forests analyses with 10‐fold and leave one subject out cross validation using all 998 and the top 30 smartphone features.

Prediction	AUC		
	10‐fold CV		LOSO CV
	998 features	Top 30 features	Top 30 features
Falls	0.94	0.88	0.79
Freezing	0.95	0.75	0.77
Postural Instability	0.90	0.91	0.79
Cognitive impairment	0.97	0.81	0.82
Difficulty doing hobbies	0.93	0.88	0.78
Need for help	0.99	0.85	0.83

Abbreviations: AUC, area under the curve, LOSO, leave one subject out and CV, cross validation.

### Comparison with other models

Logistic regression models described by Ehgoetz et al. and Velseboer et al. were applied to the clinical data from participants who had contributed smartphone recordings and the accuracy of their predictions assessed.^5^, ^9^ As a comparator, smartphone recordings from the same participants were used to train machine learning algorithms (random forests) which were validated using LOSO CV (Table 4). For the prediction of future freezing, the LOSO CV model using 30 smartphone features outperforms the logistic regression model. Although to a lesser degree, the same is true when using two smartphone features, equivalent to the number of clinical variables in the logistic regression model. Seven participants who contributed smartphone recordings went on to develop dementia (*n* = 2) and/or postural instability (*n* = 6) while at the same time having the baseline clinical variables necessary for the Velseboer et al. model. None of the participants who contributed smartphone recordings had died by their next clinic visit. For the prediction of the composite adverse outcome of dementia, postural instability, or death^9^ the AUC for the logistic regression model (0.81) was slightly higher than that garnered through a machine learning approach using the top 30 smartphone features (0.76). When creating models, the lack of any formal CV can result in over‐fitting. Indeed, when a similar approach is adopted to Ehgoetz et al., that is, when models are trained and tested on the same data set, without any splitting into independent train and test sets, inflated AUC values of 1 for both the prediction of freezing and the composite adverse outcome are obtained using a machine learning approach.

**Table 4:** Comparison with two existing logistic regression clinical prediction models for the prediction of future freezing and a composite adverse outcome of cognitive impairment, postural instability, or death.

Prediction	Method	Input variables	AUC (95% confidence intervals)^1^
Future freezing	Logistic regression model as described by Ehgoetz et al.^5^	Two clinical variables: Total FOG score and HADS score	0.56 (0.44–0.67)
	Random forests with LOSO CV	Top 2 smartphone features	0.59 (0.55–0.64)
	Random forests with LOSO CV	Top 30 smartphone features	0.77 (0.73–0.80)
Composite adverse outcome of dementia, postural instability or death^2^	Logistic regression model as described by Velseboer et al.^9^	three clinical variables: Animals named, age and axial score	0.81 (0.40–0.96)
	Random forests with LOSO CV	Top 3 smartphone features	0.63 (0.52–0.71)
	Random forests with LOSO CV	Top 30 smartphone features	0.76 (0.68–0.84)

Abbreviations: AUC, area under the curve, FOG, Freezing of gait, HADS, Hospital anxiety and depression scale, LOSO CV, leave one subject out cross validation.

1 Confidence intervals for the logistic regression model are calculated across single predictions for each set of clinical data whereas confidence intervals for LOSO CV are calculated using a bootstrapping approach; the two confidence intervals are therefore not directly comparable.

2 No participants had died by the time of their next clinic visit.

## Discussion

Prediction of disease progression and their milestones is a challenge for clinicians and patients alike. We describe for the first time the innovative use of a 7‐min smartphone test in isolation to predict future clinical and functional change in early Parkinson's. Using a simple smartphone test, we can predict the onset of disease milestones including falls, freezing, postural instability, and cognitive impairment 18 months before occurrence with high levels of accuracy. Predictions are made on an individual basis using data from smartphone tests alone, without the need for clinical examination scores or patient questionnaires. A thorough evaluation of our machine learning approach is performed using two CV schemes; (1) 10‐fold CV and (2) LOSO CV as well as (3) comparison with existing logistic regression models. AUC values exceed 0.90 for 10‐fold CV using all 998 smartphone features and 0.75 with a reduction to 30 features, using either CV scheme (see Table 1). Performance is comparable with/ significantly better than that achieved with existing prediction models which use combinations of different clinical questionnaires and assessments, requiring time and skill to administer (see Table 2).^5^, ^9^, ^19^, ^27^, ^28^

These promising early results have the potential to provide people with Parkinson's and those partnering in their clinical care with greater information about their disease course, identifying those at risk of developing a future adverse outcome. In the age of limited clinical resources and an aging population, it is conceivable that such risk stratifying tools may assist in remote healthcare delivery, allowing people with well controlled Parkinson's to be seen in person less frequently while those flagged as being at risk of an adverse outcome could be offered closer clinical surveillance. The cost effectiveness of such an approach remains to be elucidated, but those at risk of falls or postural instability could be referred for a falls risk assessment to identify proactively falls risk factors and modify these where appropriate.^29^ The earlier detection of those with freezing may also allow medication adjustments to be made or cognitive training to be considered, with potential improvements in quality of life.^30^, ^31^

### Limitations and strengths

In predicting future falls, freezing, difficulty doing hobbies, and the need for future help, we are limited by our reliance on the accuracy of self‐reported answers to subjective questionnaires, with potential recall bias. In recruiting participants diagnosed with Parkinson's in life, we are limited by the diagnostic accuracy of clinicians, itself subject to significant variability, with pooled rates of 73.8% in non‐experts rising to 83.9% in experts having refined their diagnoses over time.^32^ In screening for cognitive impairment, we chose a definition based on the transition from a total MoCA score ≥ 26, to a MoCA score < 26,^21^ in the absence of formal caregiver interviews. A MoCA score < 26 has previously been found to be associated with a sensitivity of 86% and specificity of 72% in screening for individuals with deficits on neuropsychological testing in at least two domains.^33^ Additionally, it allows classification of PD Mild Cognitive Impairment according to MDS Task Force Level I criteria,^34^ which, although subject to some temporal fluctuation, has been shown to have prognostic value in identifying patients at risk of developing future PD dementia.^35^ Although the intention was to detect individuals who may benefit from further cognitive evaluation, neuropsychological assessments were not performed in this study due to time constraints, but would be an interesting avenue of future investigation, potentially alongside the prospective evaluation of predictions in independent cohorts.

Although the numbers we report are comparable with similar studies seeking to predict future clinical outcomes, our study includes relatively small numbers of participants who develop the outcomes of interest at their next clinic visit.^5^, ^9^ Although our results are encouraging, the LOSO CV results in particular are likely to improve as we continue to accrue data. The prediction accuracies reported in this study were obtained using only a single machine learning algorithm (random forests). As we accrue more data, future studies could explore the efficacy of using a range of different state‐of‐the‐art classifiers that may require larger training samples.

Comparing our smartphone approach with existing clinical prediction models, it was necessary to make some assumptions and extrapolations. The Velseboer et al. model developed by the CARPA study and validated in CamPaIGN was trained to predict a composite adverse outcome of death, dementia and postural instability after a 5‐year period using baseline data.^9^ As participants in the Discovery study do not perform Mini‐Mental State Examination (MMSE) assessments after their third visit, we used a MoCA ≤ 20 with an IQCODE> 52 to define dementia, as opposed to the original cut off of an MMSE < 26. We excluded participants with a baseline MoCA < 26 from analysis. As none of the participants who performed smartphone assessments had died by their next clinic visit, we are unable to assess the accuracy of their model in predicting death. Given the adaptations made it is encouraging that the AUC value associated with the application of the logistic regression model to our data as well as that achieved using trained machine learning algorithms, are comparable with those published by the authors of the original study.

In contrast, we were not able to achieve an AUC comparable to the values reported by Ehgoetz et al. when we applied their logistic regression model to our clinical data. Although the individuals who went on to develop freezing at their follow up visit were of a similar number, age and sex ratio, in the Ehgoetz et al. study they were on much larger baseline levodopa equivalent doses and had significantly higher levels of baseline anxiety than those who did not develop freezing, whereas in our study such baseline differences were not observed.

Our aim has been to use smartphone devices to measure symptom severity in a way that is potentially scalable to large healthcare systems where resources are limited. To that end, we elected to collect data in both clinical and home environments without relying on cross‐device reproducibility, using a wide range of off‐the‐shelf consumer grade smartphones (manufactured by major international brands) that were equipped with a tri‐axial accelerometer (see Figure 1). Prioritising precision, the vast majority of wearable device studies have recorded data under highly controlled laboratory settings, using exactly the same (often costly) hardware and software across participants. This approach is likely to result in higher quality data, as confounding effects due to variations in the in‐built accelerometer are minimized. We would therefore expect our use of different device settings to be associated with slightly worse classification accuracies, than if the exact same smartphone model were used across the cohort. However crucially, our study is scalable as it does not depend on cross‐device reproducibility, leading to greater confidence in our interpretation of the overall classification results.

To the best of our knowledge, this is the largest study of its kind to use consumer‐grade smartphones to capture real‐world data recordings from 237 people with Parkinson's, studied longitudinally over 18 months. The Oxford Discovery cohort comprises community‐ascertained patients and as such should more faithfully recapitulate disease evolution encountered in clinical practice. This study demonstrates the tractability of simple smartphone recordings across large populations, to accurately predict future clinically relevant outcomes. With time, our goal would be to seek the registrations and approvals necessary to translate our findings into routine clinical practice, with the ultimate aim of improving the care of people with Parkinson's.

## Author Contributions

CL, SA, FB, YBS, and MH involved in study concept and design. CL involved in drafting of the manuscript; All authors involved in data acquisition, analysis, or interpretation and critical review of the manuscript.

## Conflicts of Interest

Christine Lo: Oxford Biomedical Research Centre; Siddharth Arora: University of Oxford; Fahd Baig: Oxford Discovery; Michael Lawton: Oxford Discovery; Claire El Mouden: Oxford Biomedical Research Centre; Tom Barber: Wellcome Trust Doctoral Training Fellowship; Claudio Ruffman: None; Johannes Klein: Oxford Health NIHR Biomedical Research Centre; Peter Brown: Medical Research Council (MC_UU_12024/1); Yoav Ben‐Shlomo: University of Bristol; Maarten de Vos: Oxford BRC, EPSRC, BBSRC, Roche; Michele Hu: Parkinson's UK Monument Discovery Award, Oxford BRC, University of Oxford, National Institute for Health Research, Michael J Fox Foundation, H2020 European Union, GE Healthcare, PSP Association. Consultant for Biogen and Roche Advisory Boards. Parkinson's UK: Targeting the pathological pathways to Parkinson's and Understanding the early pathological pathways in Parkinson's Disease

## Supporting information


**Figure S1** **.** Change in prediction accuracy with increase in feature number for 10‐fold and leave one subject out (LOSO) cross validation (CV).
**Table S1** **.** Description of the modelling algorithm.

## References

[1] Lawton M , Ben‐Shlomo Y , May MT , et al. Developing and validating Parkinson's disease subtypes and their motor and cognitive progression. J Neurol Neurosurg Psychiatry 2018;89:1279–1287. 30464029 10.1136/jnnp-2018-318337 PMC6288789

[2] Horváth K , Aschermann Z , Ács P , et al. Minimal clinically important difference on the motor examination part of MDS‐UPDRS. Parkinsonism Relat Disord 2015;21:1421–1426. 26578041 10.1016/j.parkreldis.2015.10.006

[3] Rascol O , Fitzer‐Attas CJ , Hauser R , et al. A double‐blind, delayed‐start trial of rasagiline in Parkinson's disease (the ADAGIO study): prespecified and post‐hoc analyses of the need for additional therapies, changes in UPDRS scores, and non‐motor outcomes. Lancet Neurol 2011;10:415–423. 21482191 10.1016/S1474-4422(11)70073-4

[4] Post B , Merkus MP , de Haan RJ , et al. Prognostic factors for the progression of Parkinson's disease: a systematic review. Movement Disord 2007;22:1839–1851. 17595026 10.1002/mds.21537

[5] Ehgoetz Martens KA , Lukasik EL , Georgiades MJ , et al. Predicting the onset of freezing of gait: A longitudinal study. Mov Disord 2018;33:128–35. 29150872 10.1002/mds.27208

[6] Williams‐Gray CH , Mason SL , Evans JR , et al. The CamPaIGN study of Parkinson's disease: 10‐year outlook in an incident population‐based cohort. J Neurol Neurosurg Psychiatry 2013;84:1258–1264. 23781007 10.1136/jnnp-2013-305277

[7] Paul SS , Canning CG , Sherrington C , et al. Three simple clinical tests to accurately predict falls in people with Parkinson's disease. Mov Disord 2013;28:655–662. 23450694 10.1002/mds.25404

[8] Duncan RP , Cavanaugh JT , Earhart GM , et al. External validation of a simple clinical tool used to predict falls in people with Parkinson disease. Parkinsonism Relat Disord. 2015;21:960–963. 26003412 10.1016/j.parkreldis.2015.05.008 PMC4509804

[9] Velseboer DC , de Bie RM , Wieske L , et al. Development and external validation of a prognostic model in newly diagnosed Parkinson disease. Neurology 2016;86:986–93. 26888991 10.1212/WNL.0000000000002437 PMC4799715

[10] Varghese J , Niewohner S , Soto‐Rey I , et al. A smart device system to identify new phenotypical characteristics in movement disorders. Front Neurol 2019;10:48. 30761078 10.3389/fneur.2019.00048 PMC6363699

[11] Barrantes S , Sanchez Egea AJ , Gonzalez Rojas HA , et al. Differential diagnosis between Parkinson's disease and essential tremor using the smartphone's accelerometer. PLoS ONE 2017;12:e0183843. 28841694 10.1371/journal.pone.0183843 PMC5571972

[12] Arora S , Venkataraman V , Zhan A , et al. Detecting and monitoring the symptoms of Parkinson's disease using smartphones: a pilot study. Parkinsonism Relat Disord 2015;21:650–653. 25819808 10.1016/j.parkreldis.2015.02.026

[13] Lipsmeier F , Taylor KI , Kilchenmann T , et al. Evaluation of smartphone‐based testing to generate exploratory outcome measures in a phase 1 Parkinson's disease clinical trial. Movement Disord 2018;33:1287–1297. 29701258 10.1002/mds.27376 PMC6175318

[14] Zhan A , Mohan S , Tarolli C , et al. Using smartphones and machine learning to quantify parkinson disease severity: the mobile parkinson disease score. JAMA Neurol 2018;75:876–880. 29582075 10.1001/jamaneurol.2018.0809 PMC5885192

[15] Szewczyk‐Krolikowski K , Tomlinson P , Nithi K , et al. The influence of age and gender on motor and non‐motor features of early Parkinson's disease: initial findings from the Oxford Parkinson Disease Center (OPDC) discovery cohort. Parkinsonism Relat Disord 2014;20:99–105. 24183678 10.1016/j.parkreldis.2013.09.025

[16] Hughes AJ , Daniel SE , Kilford L , Lees AJ . Accuracy of clinical diagnosis of idiopathic Parkinson's disease: a clinico‐pathological study of 100 cases. J Neurol Neurosurg Psychiatry 1992;55:181–184. 1564476 10.1136/jnnp.55.3.181 PMC1014720

[17] Lawton M , Baig F , Rolinski M , et al. Parkinson's disease subtypes in the Oxford Parkinson Disease Centre (OPDC) discovery cohort. J Parkinson's Dis 2015;5:269–279. 26405788 10.3233/JPD-140523 PMC4923737

[18] Arora S , Baig F , Lo C , et al. Smartphone motor testing to distinguish idiopathic REM sleep behavior disorder, controls, and PD. Neurology 2018;91:e1528–e38. 30232246 10.1212/WNL.0000000000006366 PMC6202945

[19] Giladi N , Tal J , Azulay T , et al. Validation of the freezing of gait questionnaire in patients with Parkinson's disease. Movement Disord 2009;24:655–661. 19127595 10.1002/mds.21745

[20] Hoehn MM , Yahr MD . Parkinsonism: onset, progression and mortality. Neurology 1967;17:427–442. 6067254 10.1212/wnl.17.5.427

[21] Hu MT , Szewczyk‐Krolikowski K , Tomlinson P , et al. Predictors of cognitive impairment in an early stage Parkinson's disease cohort. Movement Disord 2014;29:351–359. 24395708 10.1002/mds.25748 PMC4235340

[22] Nasreddine ZS , Phillips NA , Bedirian V , et al. The Montreal Cognitive Assessment, MoCA: a brief screening tool for mild cognitive impairment. J Am Geriatr Soc 2005;53:695–699. 15817019 10.1111/j.1532-5415.2005.53221.x

[23] Goetz CG , Tilley BC , Shaftman SR , et al. Movement disorder society‐sponsored revision of the Unified Parkinson's Disease rating scale (MDS‐UPDRS): scale presentation and clinimetric testing results. Movement Disord 2008;23:2129–2170. 19025984 10.1002/mds.22340

[24] Arora S , Baig F , Lo C , et al. Smartphone motor testing to distinguish idiopathic REM sleep behaviour disorder, controls and PD. Neurology 2018;91:e1528–e1538. 30232246 10.1212/WNL.0000000000006366 PMC6202945

[25] Breiman L . Random forests. Mach Learn 2001;45:5–32.

[26] Harrison JK , Stott DJ , McShane R . et al. Informant Questionnaire on Cognitive Decline in the Elderly (IQCODE) for the early diagnosis of dementia across a variety of healthcare settings. Cochrane Database Syst Rev. 2016;11:CD011333. 27869298 10.1002/14651858.CD011333.pub2 PMC6477966

[27] Zigmond AS , Snaith RP . The hospital anxiety and depression scale. Acta Psychiatr Scand 1983;67:361–370. 6880820 10.1111/j.1600-0447.1983.tb09716.x

[28] Fahn S , Elton RL . UPDRS program members . Unified Parkinsons Disease Rating Scale In: Fahn S., Marsden C. D., Goldstein M., Calne D. B. eds. Recent developments in Parkinsons disease. Florham Park, NJ: Macmillan Healthcare Information, 1987.

[29] Fasano A , Canning CG , Hausdorff JM , et al. Falls in Parkinson's disease: A complex and evolving picture. Movement Disord 2017;32:1524–1536. 29067726 10.1002/mds.27195

[30] Walton CC , Mowszowski L , Gilat M , et al. Cognitive training for freezing of gait in Parkinson's disease: a randomized controlled trial. NPJ Parkinson's Dis. 2018;4:15. 29796409 10.1038/s41531-018-0052-6 PMC5959878

[31] Perez‐Lloret S , Negre‐Pages L , Damier P , et al. Prevalence, determinants, and effect on quality of life of freezing of gait in parkinson disease. JAMA Neurol 2014;71:884–890. 24839938 10.1001/jamaneurol.2014.753

[32] Rizzo G , Copetti M , Arcuti S , et al. Accuracy of clinical diagnosis of Parkinson disease: a systematic review and meta‐analysis. Neurology 2016;86:566–576. 26764028 10.1212/WNL.0000000000002350

[33] Hoops S , Nazem S , Siderowf AD , et al. Validity of the MoCA and MMSE in the detection of MCI and dementia in Parkinson disease. Neurology 2009;73:1738–1745. 19933974 10.1212/WNL.0b013e3181c34b47 PMC2788810

[34] Litvan I , Goldman JG , Troster AI , et al. Diagnostic criteria for mild cognitive impairment in Parkinson's disease: Movement Disorder Society Task Force guidelines. Mov Disord 2012;27:349–356. 22275317 10.1002/mds.24893 PMC3641655

[35] Lawson RA , Yarnall AJ , Duncan GW , et al. Stability of mild cognitive impairment in newly diagnosed Parkinson's disease. J Neurol Neurosurg Psychiatry 2017;88:648–652. 28250029 10.1136/jnnp-2016-315099 PMC5537517

