# Supplementary material for: Predicting motor, cognitive & functional impairment in Parkinson's
Source: Ann Clin Transl Neurol. 2019 Jul 26;6(8):1498–509. doi: 10.1002/acn3.50853 (PMC6689691; doi:10.1002/acn3.50853)
Supplement: Supplementary file 1 — Figure S1 . Change in prediction accuracy with increase in feature number for 10‐fold and leave one subject out (LOSO) cross validation (CV). Table S1 . Description of the modelling algorithm. [file ACN3-6-1498-s001.docx]

Supplementary material

Supplementary Figure 1 | **The change in prediction accuracy with increase in feature number for 10-fold and leave one subject out (LOSO) cross validation (CV)** Abbreviation: AUC = area under the curve.

Supplementary Table 1 | **Description of the modelling algorithm** Abbreviation: CV = cross validation.

| ***Algorithm: Predicting outcomes in Parkinson’s*** |
| --- |
| ***Input:*** $\boldsymbol{x}_{\boldsymbol{1}}$***,*** $\boldsymbol{x}_{\boldsymbol{2}}$***, … ,***$\boldsymbol{x}_{\boldsymbol{998}}$ (998 smartphone *features*)  *N - Number of classification trees in the random forest*  *M - Number of predictor variables to be used for random feature selection*  ***Output:***$\boldsymbol{y}$ (clinical outcome of interest) |
| ***Step 1: Pre-processing (Ignore the recording if data for any of the 7 tasks is missing, and extract 998 features from each complete recording)***  ***for i = 1:1:total_no_recordings***  ***for j = 1:1:7***  ***if (isempty(smartphone_rec(i,j))***  ***ignore smartphone_rec(i,1: 7)***  ***else***  ***Features(i,1:998) = ExtractFeat(smartphone_rec(i,1:7))***  ***end***  ***end***  ***end*** |
| ***Step 2: Feature Selection***   1. ***Balance data (undersample majority class)*** 2. ***Split data into train and test sets (for a given CV scheme)*** 3. ***Use random forests to get feature ranking (using only the training sample)*** 4. ***Repeat above process k number of times*** 5. ***Obtain one final set of feature rankings using majority voting*** |
| ***Step 3: Classification and Evaluation***   1. ***Balance data (undersample majority class)*** 2. ***Split data into train and test sets (for a given CV scheme)*** 3. ***Use 10-fold and leave one subject out cross validation (train data to learn model, test data to evaluate prediction)*** 4. ***Repeat above process k number of times times*** 5. ***Evaluate classifier (compute sensitivity and specificity)*** |
